# Cytosine methylation regulates DNA bendability depending on the curvature†

**DOI:** 10.1039/d1sc07115g

**Published:** 2022-06-02

**Authors:** Sanghun Yeou, Jihee Hwang, Jaehun Yi, Cheolhee Kim, Seong Keun Kim, Nam Ki Lee

**Affiliations:** Department of Chemistry, Seoul National University 08832 Seoul Republic of Korea namkilee@snu.ac.kr; National Science Museum Daejeon 34143 Republic of Korea

## Abstract

Cytosine methylation plays an essential role in many biological processes, such as nucleosome inactivation and regulation of gene expression. The modulation of DNA mechanics may be one of the regulatory mechanisms influenced by cytosine methylation. However, it remains unclear how methylation influences DNA mechanics. Here, we show that methylation has contrasting effects on the bending property of dsDNA depending on DNA curvature. We directly applied bending force on 30 base pairs of dsDNA using a D-shaped DNA nanostructure and measured the degree of bending using single-molecule fluorescence resonance energy transfer without surface immobilization. When dsDNA is weakly bent, methylation increases the stiffness of dsDNA. The stiffness of dsDNA increased by approximately 8% with a single methylation site for 30 bp dsDNA. When dsDNA is highly bent by a strong force, it forms a kink, *i.e.*, a sharp bending of dsDNA. Under strong bending, methylation destabilizes the non-kink form compared with the kink form, which makes dsDNA near the kink region apparently more bendable. However, if the kink region is methylated, the kink form is destabilized, and dsDNA becomes stiffer. As a result, methylation increases the stiffness of weakly bent dsDNA and concurrently can promote kink formation, which may stabilize the nucleosome structure. Our results provide new insight into the effect of methylation, showing that cytosine methylation has opposite effects on DNA mechanics depending on its curvature and methylation location.

## Abstract 2

D-shaped DNA is used to observe dsDNA bending mechanics. Cytosine methylation increases the intrinsic stiffness of dsDNA. Under strong bending, methylation stabilizes or destabilizes a kink form depending on methylation sites.

## Graphical abstract

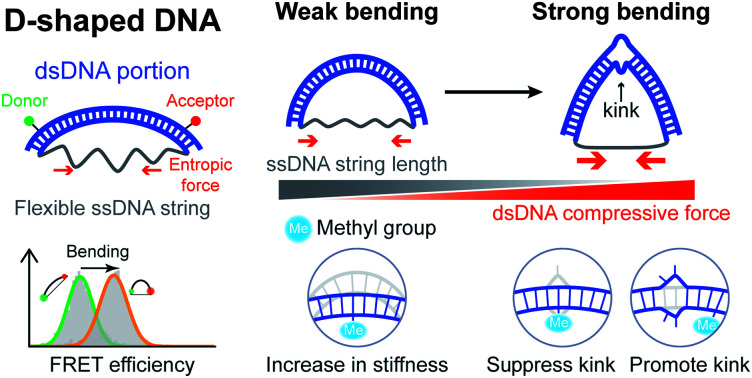

## Introduction

DNA methylation is one of the most vital epigenetic modifications of cytosine.^1^ It replaces a hydrogen atom of the fifth carbon of cytosine with a methyl group (5-methylcytosine, 5-mC), which can be found in most animals and plants.^2^ Although methylation does not alter DNA sequences, it regulates a variety of phenomena, such as inactivation of the X chromosome,^3^ cellular differentiation,^4^ and gene expression.^5^ CpG site methylation plays a pivotal role in regulatory functions,^6,7^ but recent results have shown that non-CpG sites can also be methylated to play a regulatory role in gene expression.^8–10^

It has long been suggested that methylation modulates the bending properties of double-stranded DNA (dsDNA), which would be one of the regulatory mechanisms governed by methylation.^11–13^ For example, the formation of nucleosomes, consisting of 147 base pairs (bp) of DNA wrapping around an 8.5 nm-diameter histone octamer, is highly related to the DNA bending structure.^14^ CpG methylation facilitates nucleosome assembly,^15^ and nucleosome occupancy is positively related to methylated CpG islands.^16^ Thus, methylation may facilitate nucleosome formation and stabilize nucleosome structure. However, the functions of methylation in the stability of nucleosomes and chromatin remodeling are still unclear.^17^

Recently, due to the importance of the effect of methylation on nucleosome structure, the modulation of dsDNA bendability by methylation has been extensively studied.^18–25^ The increment of the looping time of methylation-mediated dsDNA, *i.e.*, the increase in the stiffness of dsDNA, was observed by DNA cyclization kinetic measurements.^18,19^ In line with these results, an increase in the persistence length of dsDNA was observed by atomic force microscopy (AFM).^20,21^ In contrast, optical and magnetic tweezer studies showed that weakly stretched methylated dsDNA has a shorter end-to-end distance than unmethylated dsDNA, which indicates a decrease in DNA stiffness.^22–25^ However, the tweezer studies also observed no change in the DNA stiffness when dsDNAs had approximately 50% GC content.^23,24^ The decrease in DNA stiffness with methylation may explain the stabilization of nucleosomes for their inactivation. However, tweezer measurements have limitations with the use of relatively long DNA and the measurement of the entropic elasticity of dsDNA with a low force, not directly dsDNA bending. The measurement of DNA looping time has an advantage with the use of a short dsDNA (<100 bp), for which curvature corresponds to the curvature of DNA in nucleosomes. However, its results appear to be opposite of the results obtained for the stabilization of nucleosomes by methylation.^18,19^ As a result, it is still controversial whether methylation increases or decreases DNA stiffness.

More importantly, none of the previous experimental work has directly observed the bending of short DNA by applying various compressive forces to measure DNA stiffness.

**Fig. 1:**
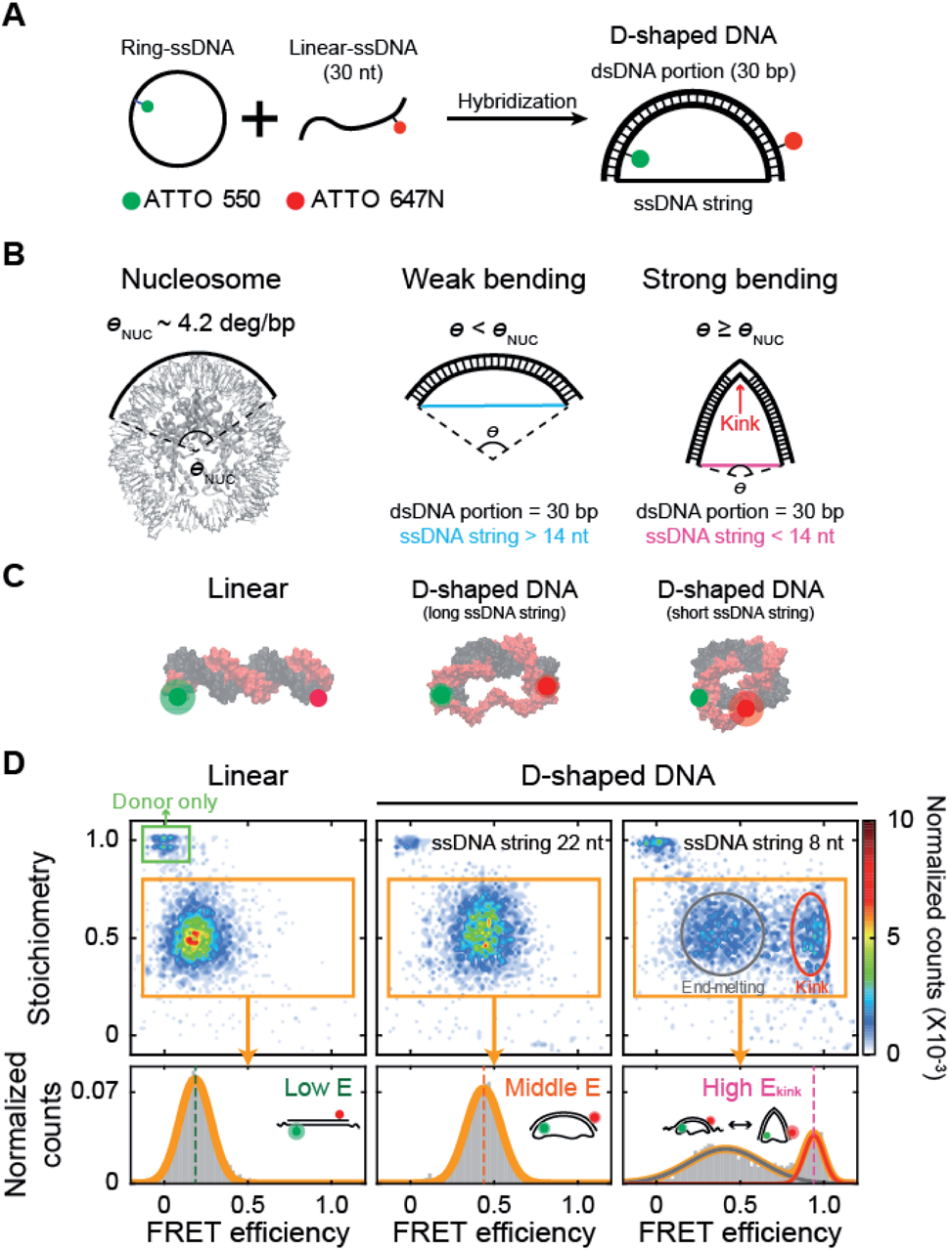
Variation in DNA curvature using D-shaped DNA and single-molecule FRET measurement. (A) Schematic description of the D-shaped DNA nanostructure. D-shaped DNA is formed by hybridization of ATTO 550-labeled ring ssDNA and ATTO 647N-labeled 30 nt linear ssDNA. D-shaped DNA consists of two parts: a rigid dsDNA portion (30 bp) and a flexible ssDNA string. The curvature of the dsDNA portion is controlled by changing the length of the ssDNA string. (B) Illustrations of DNA samples according to curvature. The curvature of nucleosomal DNA (*θ*_NUC_) is approximately 4.2° per bp (nucleosome image from PDB ID 6KVD).^26^ D-shaped DNA with ssDNA string longer than 14 nt has weak bending, and its curvature is lower than the curvature of nucleosomal DNA. D-shaped DNA with ssDNA string shorter than 14 nt has strong bending, and its curvature is higher than the curvature of nucleosomal DNA. A kink occurs on the dsDNA portion under strong bending conditions. (C) Illustrations of linear DNA, D-shaped DNA with a long ssDNA string, and D-shaped DNA with a short ssDNA string. (D) Single-molecule FRET measurement of D-shaped DNA. 2D FRET histograms of linear DNA, D-shaped DNA with a 22 nt ssDNA string (D30-S22), and D-shaped DNA with an 8 nt ssDNA string (D30-S8) (the burst counts were 4120, 3236, and 3350, respectively). The single-molecule bursts in the green box indicate the donor-only species. The bursts in the orange boxes indicate the dual-labeled D-shaped DNAs, which were used to obtain 1D FRET efficiency histograms in the bottom panel. D30-S8 shows two states of a kink form and an end-melting form. The FRET efficiency was obtained by fitting the 1D histograms using single or two Gaussian distributions.

Here, we report that methylation has contrasting effects on the bending properties of dsDNA depending on the DNA curvature using D-shaped DNA (Fig. 1A)^27–32^ and single molecule fluorescence resonance energy transfer (smFRET, Fig. S1†).^33^ We show that when the curvature of dsDNA is smaller than the nucleosome curvature, *i.e.*, weak bending (Fig. 1B), methylation increases the stiffness of dsDNA. When the bending of dsDNA increases to be comparable to or larger than the nucleosome curvature, *i.e.*, strong bending (Fig. 1B), dsDNA shows a deformed structure of a kink, *i.e.*, a sharp bending of dsDNA with flexible denatured local base pairs at the center of dsDNA.^29–32,34,35^ In kinked dsDNA, methylation destabilizes non-kink form compared with a kink form and thus, dsDNA becomes easily bendable using the kink as a hinge. However, when the cytosine in the kink region is methylated, the kink form is destabilized, and dsDNA becomes stiffer.

As a result, our study demonstrates that methylation can have a different effect on DNA mechanics depending on its curvature and provides a primary clue to understand the previous observations that methylation increases DNA stiffness while stabilizing nucleosome structure.^36,37^

## Results

### Modulation of the DNA bending using D-shaped DNA

We investigated the bending properties of methylated dsDNA using a D-shaped DNA nanostructure (Fig. 1A).^27–31^ The curvature of 30 bp dsDNA was controlled by using D-shaped DNA, which consisted of ssDNA ring and partially complementary linear 30 nt ssDNA. By varying the length of the ssDNA ring, we applied different forces on dsDNA portion and thus modulated the curvatures of methylated and unmethylated dsDNAs. D-shaped DNA has two different parts: a rigid dsDNA and a flexible ssDNA string. For example, if a 52 nt ssDNA ring is used, the lengths of the dsDNA portion and the ssDNA string are 30 bp and 22 nt, respectively. For convenience, we named this D-shaped DNA D30-S22, *i.e.*, the dsDNA portion was 30 bp, and the length of the ssDNA string was 22 nt. The entropic stretching force, which depends on the ssDNA string length, bends the dsDNA portion.^27,28^ The 30 bp dsDNA portion bends smoothly when the length of the ssDNA string is longer than 14 nt.^30,31^ However, if the length of the ssDNA string is shorter than 14 nt, the ssDNA string strongly compresses the dsDNA portion. Highly bent dsDNA must release its bending stress. One way is to denature local base pairs at the center of dsDNA, and then dsDNA has a highly bent structure, which is a kink (Fig. 1B).^29–31,34^ In our previous work, we showed that the kink in the 30 bp dsDNA portion is generated when the length of the ssDNA string is shorter than 14 nt.^31^ In this manner, we modulated the curvature of the dsDNA portion at will (Fig. 1C).

Then, the degree of bending curvature was monitored by observing the end-to-end distance of the dsDNA portion using smFRET. The end-to-end distance of the dsDNA portion was used to distinguish the states of dsDNA, such as linear, weak bending, strong bending, and kink formation. Each end of the dsDNA portion of D-shaped DNA (4^th^ and 27^th^ base pairs of dsDNA portion) was labeled with ATTO 550 and ATTO 647N as a FRET donor and acceptor, respectively (Fig. 1C). The FRET efficiency of each D-shaped DNA was measured using the alternating laser excitation method (ALEX) at the salt concentration close to the physiological conditions and no surface tethering (Fig. S1†).^38–41^ When a sample passed through the confocal volume, a burst of fluorescence signal was detected instantaneously. Then, the FRET efficiency and stoichiometry of each molecule were calculated and represented as a two-dimensional diagram (Fig. 1D and S1†). The FRET efficiency represents the distance between donor and acceptor dyes, while stoichiometry reports the number of donor and acceptor dyes in a molecule. We used the stoichiometry parameter to select only the D-shaped DNA molecules (Fig. 1D, the orange box).

As smFRET has a high dye-to-dye distance sensitivity, it can detect small changes in the bending curvature of dsDNA.^40^ The average FRET efficiency (*E*) of a linear 30 bp dsDNA was approximately 0.18 (Fig. 1D, the left panel), while that of smoothly bent dsDNA with a 22 nt ssDNA string (D30-S22) was 0.43 (Fig. 1D, the middle panel). The FRET efficiency distribution of D-shaped DNA with an 8 nt ssDNA string (D30-S8) showed two states (Fig. 1D, the right panel): the high FRET efficiency conformer represents a kink form, and the low FRET efficiency conformer is an end-melting form with weak bending.^31^ Because the kink induces sharp bending of the dsDNA portion, the average FRET efficiency of the kink (*E*_kink_) form was as high as 0.95, which is much higher than the FRET efficiency of the weakly bent dsDNA.

### Cytosine methylation increases the intrinsic stiffness of dsDNA with a low curvature

**Fig. 2:**
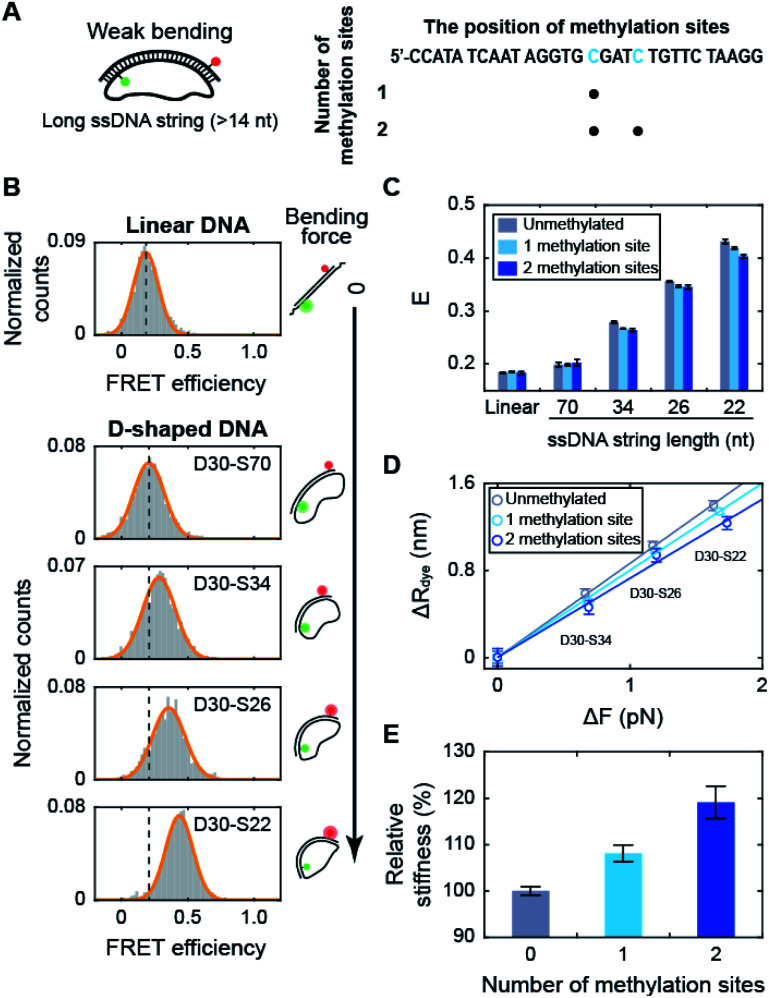
The effect of methylation on dsDNA stiffness with a low curvature. (A) Schematic representation of D-shaped DNA with a low curvature (weak bending). D-shaped DNAs with ssDNA string length longer than 14 nt were used. The methylation sites on the dsDNA portion are shown in blue in the right panel. (B) 1D FRET histograms for linear DNA and D-shaped DNAs. The length of the ssDNA string was gradually reduced from 70 nt to 22 nt (D30-S70, D30-S34, D30-S26, and D30-S22). The histograms were fitted to a single Gaussian distribution. (C) The average FRET efficiencies (E) of the linear and D-shaped DNAs. The gray bars denote unmethylated sequences, and the light blue and blue bars represent one copy and two copies of methylation, respectively. FRET histograms are presented in (B) and S2.† The error bars were obtained from three independent measurements. (D) The reduced end-to-end distances of the dsDNA portion (Δ*R*) as a function of the compressive force (Δ*F*_bend_), which were calculated from (C). The linear approximations depending on the number of methylation sites are shown as solid lines: unmethylated sequence (the gray line, where *y* = 0.86 × *x*), one copy of methylation site (the light blue line, where *y* = 0.80 × *x*) and two copies of methylation sites (the blue line, where *y* = 0.73 × *x*). (E) The relative stiffness of dsDNA depending on the number of methylation sites, calculated from the data in (D).

Using D-shaped DNA, we investigated the effect of methylation on the stiffness of weakly bent dsDNA (Fig. 2A). We first measured the FRET efficiencies of D-shaped DNAs as the length of the ssDNA string decreased (Fig. 2B). As the ssDNA string length was shortened from 70 nt to 22 nt (D30-S70, D30-S34, D30-S26, and D30-S22), *i.e.*, increasing the bending force on the dsDNA portion, the FRET efficiency of D-shaped DNA gradually increased (Fig. 2B). The increase in the FRET efficiency denotes the decrease in the dye-to-dye distance in the dsDNA portion by bending. Then, we placed one or two methylation sites on the dsDNA portion (Fig. 2A and S2†). Methylation had no effect on the FRET efficiency of the linear dsDNA (Fig. 2C and S2†). D-shaped DNA with a 70 nt string length also showed a negligible change in FRET efficiency with methylation (Fig. 2C). However, as the string length was reduced from 34 nt to 22 nt, the FRET efficiencies of D-shaped DNAs decreased with methylation. Two methylation sites decreased the FRET efficiencies of D-shaped DNAs more than one methylation site (Fig. 2C). Although the FRET histogram is broad, the average FRET efficiency obtained from Gaussian fitting is pretty accurate.^19,42,43^ Statistically, the error of the center position (FRET efficiency) was calculated to be 0.002.^44^ This value is similar with our experimental results (SEM = 0.004), obtained from three repeated measurements. In our measurement, the FRET efficiency changed approximately 0.03 by methylation, which is much larger than the error bar obtained by both Gaussian fitting and experimental results. The lower bending of dsDNA by methylation indicates that cytosine methylation increases the intrinsic stiffness of DNA with a low curvature.

When we used the high salt concentration (1 M NaCl), methylation again decreased the FRET efficiency of D30-S22 (Fig. S3†). However, the variation of the FRET efficiency by methylation at the high salt condition was smaller than that at the physiological salt condition. Thus, the effect of methylation on DNA stiffness seems to be diminished at high salt condition. Then, we tested the effect of methylation locations on DNA stiffness. When we put two methylation sites at the lateral region, the same amount of decrease in the FRET efficiency was observed (Fig. S4†). Thus, the increase in DNA stiffness by methylation was not the effect of the different methylation locations.

Next, we tested the methylation effect on dsDNA stiffness with different CG ratios (Fig. S5†). The *E* value of D30-S22 decreased as the number of methylated sites increased for three dsDNAs with different CG ratios. These results indicate that methylation increases DNA stiffness regardless of the CG ratio. However, as the CG ratio becomes higher, the effect of methylation on DNA stiffness is decreased.^45^ These results imply that methylation increases the stiffness of dsDNA under a weak bending force regardless of the methylation locations, sequences, CG ratios, and salt concentration.

Using the distance variation with methylation, we calculated the relative stiffness of dsDNA depending on the number of methylation sites (Fig. 2D and E; see ESI for more details on the calculations†).^31,46,47^ The intrinsic stiffness of dsDNA has been described by the worm-like chain model.^48^ Using D-shaped DNA with a 70 nt ssDNA string, where DNA bending begins, as a reference, the bending force increment (Δ*F*_bend_) and the reduced end-to-end distance (Δ*R*) of dsDNA portion were estimated (Fig. 2D). The slope of the linear approximation in Fig. 2D was inversely proportional to the stiffness of dsDNA (Fig. 2E). As a result, the stiffness of dsDNA increased by approximately 8% with a single methylation site and 19% with two methylation sites for 30 bp dsDNA. The number of CpG sites in the λ-phage genome varied from 7 to 39 per 500 bp.^23^ If all CpG sites are methylated, methylation will increase the stiffness of λ-phage DNA by approximately 2.5–23%.

### Methylation increases the population of the kink form of dsDNA with a high curvature

Next, we investigated the effect of methylation on DNA bending with a high curvature. When the ssDNA string length is shorter than 14 nt, D-shaped DNA forms two different structures consisting of a kink and a bent form with a few base pairs melting at the ends of the dsDNA portion (end melting).^31^ Because the kink form has locally denatured base pairs, dsDNA with a kink has a high flexibility.

**Fig. 3:**
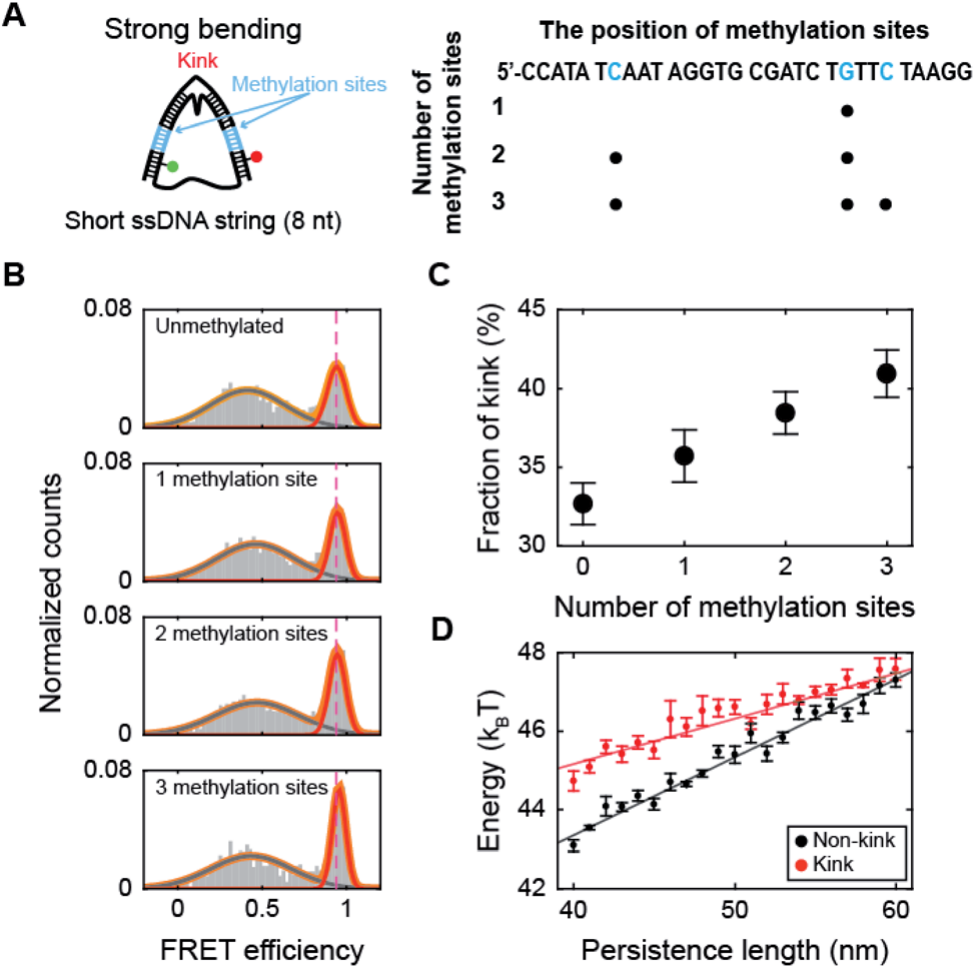
The effect of methylation at the non-kink region of the dsDNA portion under a strong bending force. (A) Schematic representation of D-shaped DNA with a high curvature (under a strong bending force). D-shaped DNA with an 8 nt ssDNA string (D30-S8) was methylated. The non-kink region of the dsDNA portion was methylated and is marked in blue. (B) 1D FRET histograms of D30-S8 depending on the number of methylation sites. The 1D histograms were fitted to two Gaussian distributions. The high FRET conformer at 0.95 denotes a kink. (C) The fraction of a kink form depending on the number of methylations. The data were obtained from (B). The error bars were obtained from three independent measurements. (D) The average bending energies of non-kink and kink dsDNA portions as a function of the persistence length of dsDNA. The energy gap between the non-kink and kink forms gradually decreases as the persistence length of dsDNA increases. The error bars were obtained from five independent simulations.

We prepared D-shaped DNA with an 8 nt ssDNA string (D30-S8) with and without methylation (Fig. 3A). The methylation sites were selected not to overlap with the kink site, *i.e.*, at the center of the dsDNA (Fig. 3A). Fig. 3B presents the FRET distribution of D30-S8 with different numbers of methylations. The FRET distribution clearly shows two populations with a kink and end melting. The most apparent effect of methylation on D30-S8 was the increase in the subpopulation of a kink form (a high FRET conformer) as the number of methylations increased (Fig. 3B and C).

It is not straightforward to explain how methylation, which increases the stiffness of weakly bent dsDNA (Fig. 2), increases the population of a kink. Thus, to understand the relationship between the intrinsic stiffness of dsDNA and the stability of the kink form, we calculated the bending energy of D-shaped DNA D30-S8 with and without a kink using a Monte Carlo simulation.^49^ The energy of the equilibrium state profile of a semiflexible loop was calculated (Fig. S6A†).^50–52^ At the equilibrium state of a kink form, the dye-to-dye distance (4^th^ and 27^th^ base pairs) was not changed by the increase in the dsDNA stiffness in the simulation (Fig. S6B†), which is in line with the experimental results for the FRET efficiencies of kink forms (Fig. S6C†). The dsDNA bending energies for both the kink and non-kink forms arise as the persistence length of dsDNA increases (Fig. 3D). This means that both kink and non-kink forms become less stable with increasing dsDNA persistence length. However, the bending energy of the non-kink form increases more rapidly than that of the kink form (Fig. 3D). This implies that the kink form becomes relatively more stable than the non-kink form as the dsDNA persistence length increases. As a result, the population of the kink form increases compared with that of the non-kink form. This simulation result explains our observation that the subpopulation of the kink form increases as the stiffness of dsDNA is increased by methylation, as shown in Fig. 3B. In addition, the increase in the local stiffness on the non-kinked region of the dsDNA portion increases the curvature of the kinked region of the dsDNA portion and the probability of sharp bending of the dsDNA portion (Fig. S7†). This result is also consistent with the finding that the increase in local stiffness due to methylation enhances the population of the kink form (Fig. 3C). Increasing the population of the kink form compared with the non-kink form by methylation may explain nucleosome structure stabilization by methylation.^36,37^

### Methylation on the kink region of dsDNA hinders kink formation

**Fig. 4:**
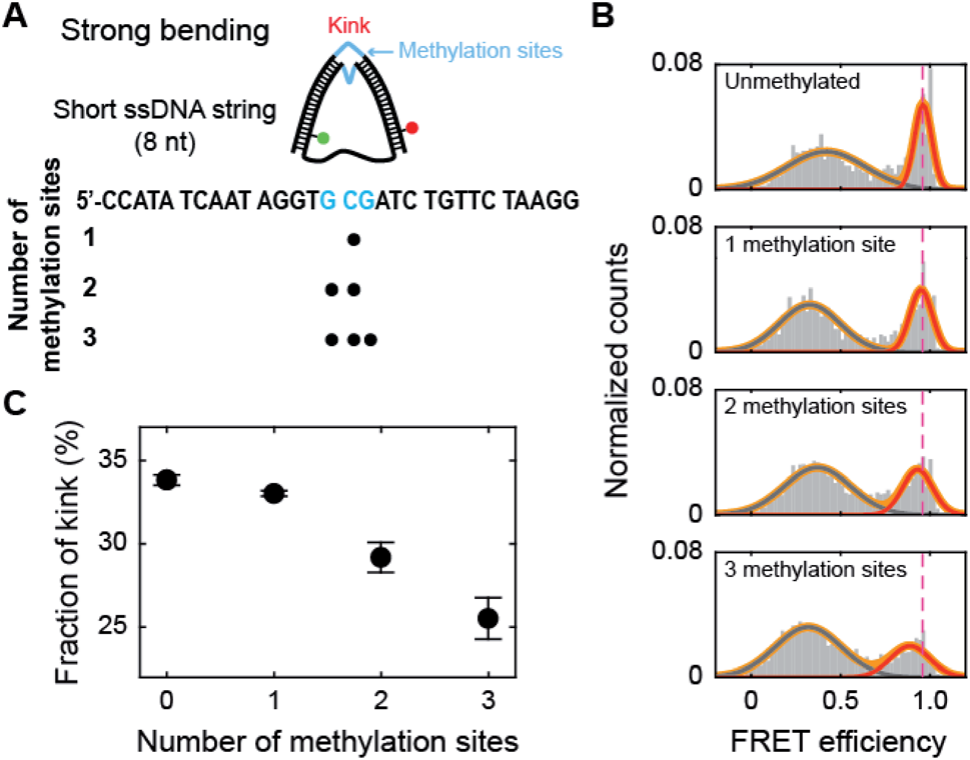
The effect of methylation at the kink region of the dsDNA portion under a strong bending force. (A) Schematic representation of D-shaped DNA with a high curvature (under a strong bending force). D-shaped DNA with an 8 nt ssDNA string (D30-S8) was methylated. The kink region of the dsDNA portion was methylated and is marked in blue. (B) 1D FRET histograms of D30-S8 depending on the number of methylation sites at the kink region. The 1D histograms were fitted to two Gaussian distributions. The high FRET conformer denotes a kink. (C) The fraction of a kink form depending on the number of methylations. The data were obtained from (B). The error bars were obtained from three independent measurements.

Methylation may occur not only in the smoothly bending region but also in the kink region of the nucleosome. Thus, we methylated the cytosine bases in the kink region, which is located at the central region of the dsDNA portion of D30-S8 (Fig. 4A).^30,31^ Interestingly, methylation decreased the subpopulation of the kink form (Fig. 4B and C), which is in sharp contrast to the trend of methylation in the non-kink region in Fig. 3. As a result, methylation of the kink region makes dsDNA stiffer. Our results show that methylation has opposite effects on the stability of a kink form, depending on the methylation regions.

**Fig. 5:**
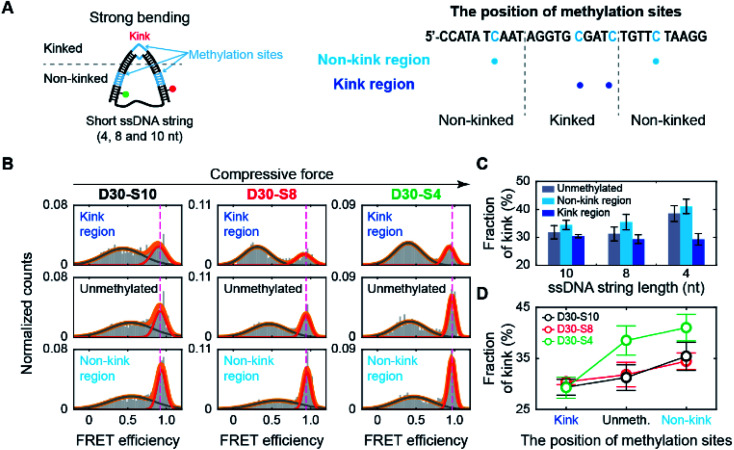
The effect of methylation at the non-kink and kink regions of the dsDNA portion under different bending forces. (A) Schematic representation of D-shaped DNA under various strong bending forces. D-shaped DNAs with 4 nt, 8 nt, and 10 nt ssDNA strings (D30-S4, D30-S8, and D30-S10) were methylated. We prepared three samples for each D-shaped DNA, *i.e.*, unmethylated, two methylation sites in the non-kink region, and two methylation sites in the kink region. The methylation sites are marked by the blue color in the right panel. (B) 1D FRET histograms of D30-S10, D30-S8, and D30-S4 depending on the region of methylation site, as shown in (A). The 1D histograms were fitted to two Gaussian distributions. The bending force of dsDNA portion is increased depending on ssDNA string length. (C) The fraction of a kink form depending on the length of the ssDNA string. The data were obtained from (B). The error bars were obtained from three independent measurements. (D) Comparison of the fraction of a kink form depending on the methylation sites.

Then, we tested whether the opposite effects of methylation on the kink form are dependent on the compressive force. We prepared D-shaped DNA with various ssDNA string lengths (4 nt, 8 nt, and 10 nt) (Fig. 5A). The non-kink region and the kink region were doubly methylated (Fig. 5A, the right panel). We methylated cytosine bases at different locations compared with the D-shaped DNA D30-S8 in Fig. 3 and 4 to determine the methylation location effect. It is clear that methylation in the non-kink region increases the subpopulation of kink forms compared with unmethylated DNA, which is independent of the length of the ssDNA string (Fig. 5B and C). When the kink region was methylated, the population of the kink decreased for all D-shaped DNAs (Fig. 5B and C). These results demonstrate that the opposite effects of methylation on the stability of the kink form are independent of the length of the ssDNA string, *i.e.*, the compressive force. Thus, it is clear that for kinked dsDNA under a strong bending force, methylation destabilizes the non-kink form compared with the kink form and thus dsDNA becomes apparently more flexible using the kink as a hinge. However, if the cytosine in the kink region is methylated, the kink form is further destabilized, and dsDNA becomes stiffer.

### Methylation on the kink region stabilizes base pairs of dsDNA

**Fig. 6:**
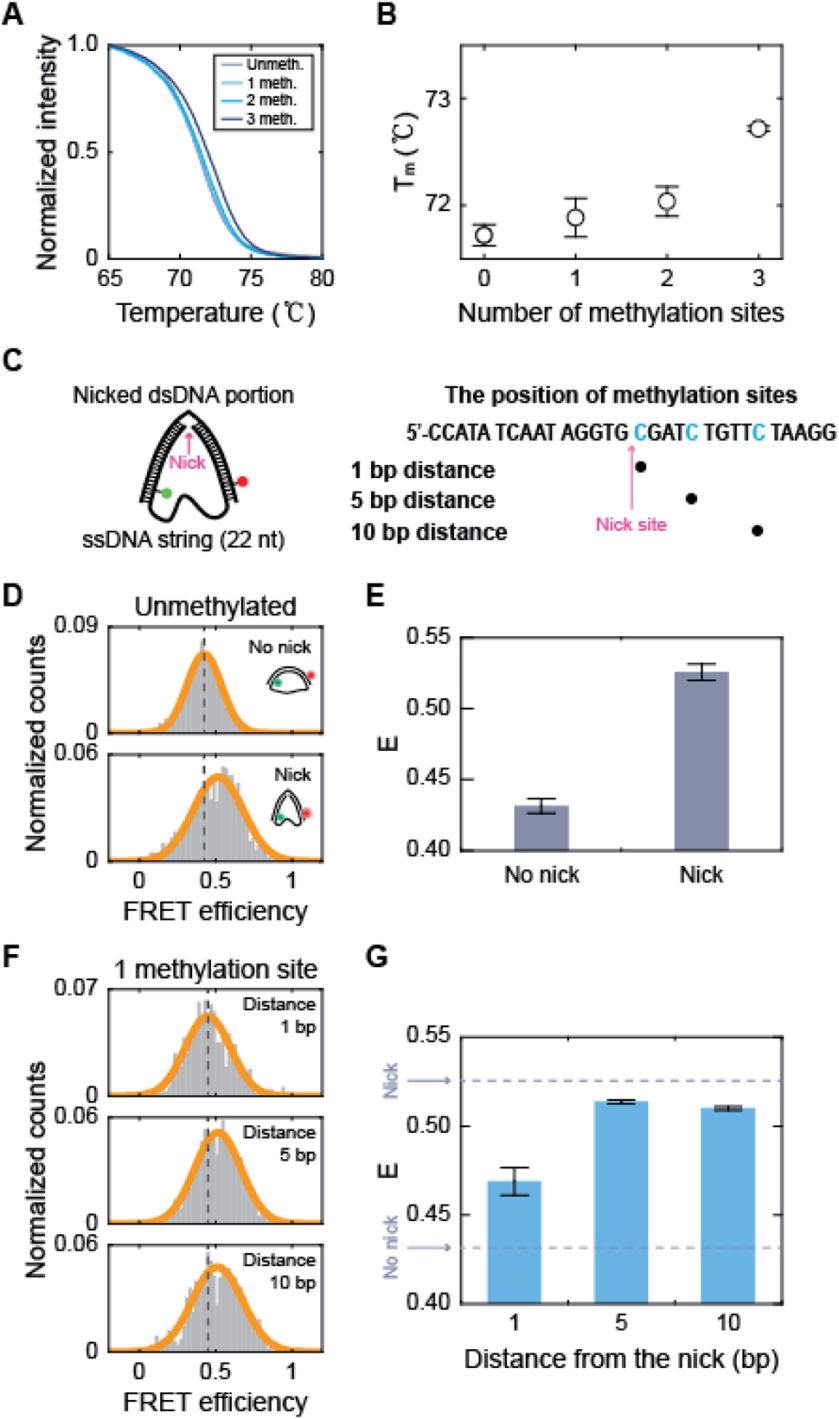
The effects of methylation on the dsDNA melting temperature and the stability of nicked D-shaped DNA. (A) Normalized fluorescence intensity of dsDNA stained by EvaGreen depending on temperature. The positions of methylation sites in each construct are the same as described in Fig. 4A. (B) Melting temperature of dsDNA depending on the number of methylation sites. The error bars were obtained from three independent measurements. (C) Schematic representation of the nicked D-shaped DNA sample. D-shaped DNA with a 22 nt ssDNA string (D30-S22) with a nick placed at the center of the dsDNA portion was used for this measurement. The methylation sites on the dsDNA portion are shown in blue (the right panel). A nick is positioned between the 15^th^ base pair and the 16^th^ base pair of the dsDNA portion. (D) 1D FRET histograms of unmethylated D30-S22 with and without a nick. The histograms were fitted to a single Gaussian distribution. (E) The average FRET efficiencies (*E*) of unmethylated D-shaped DNA with and without a nick from (D). The FRET efficiencies of D30-S22 with and without a nick were 0.53 and 0.43, respectively. The error bars were obtained from three independent measurements. (F) 1D FRET histograms for nicked D-shaped DNA with a single methylation site at 1 bp, 5 bp, and 10 bp distances from the nick site. The histograms were fitted to a single Gaussian distribution. (G) *E* depending on the distance between the nick site and methylation base pair. The error bars were obtained from three independent measurements.

How does methylation on the kink region decrease the population of the kink form? It has been shown that the melting temperature of dsDNA increases as the amount of 5-mC increases.^53^ Measurements using optical and magnetic tweezers also showed that cytosine methylation inhibits unwinding-induced melting of base pairs,^25^ and simulation studies demonstrated that methylation increases stacking and base pairing interaction energy.^19,54^ Thus, we suspected that methylation in the kink region may stabilize base pairing and prevent denaturation in the kink region, which will deter kink formation. We first measured the melting temperature (*T*_m_) of 30 bp linear dsDNA with the same sequences we used for D-shaped DNA in Fig. 4A.^55^ The *T*_m_ of dsDNA increased as the number of methylated sites increases (Fig. 6A and B). The *T*_m_ increment was also observed for the methylation at different locations (Fig. S8†), which is in line with previous work.^53^ This result suggests that cytosine methylation seems to stabilize base pairs to prevent denaturation and thus inhibits kink formation, as shown in Fig. 4.

To further test our explanation, we prepared D-shaped DNAs with a nick, *i.e.*, a break in a single-stranded DNA, at the center of the dsDNA portion (Fig. 6C). It has been reported that nicked dsDNA is more bendable than normal dsDNA because the bending force easily induces the disruption (loss of base stacking) on the nick site due to the lack of structural support provided by the phosphate backbone.^32,56–58^ Indeed, the FRET efficiency of D-shaped DNA D30-S22 (weak bending) was 0.43, but it increased to 0.53 with the nick (Fig. 6D and E). Then, we placed 5-mC at 1 bp, 5 bp, and 10 bp away from the nick site (Fig. 6C, the right panel). The D-shaped DNAs with a single methylation site at a distance from the nick (5 bp and 10 bp distance) showed FRET efficiency similar to that of the unmethylated sample (Fig. 6F and G). However, when a single methylation site was positioned right beside the nick (1 bp distance), the FRET efficiency of D-shaped DNA, 0.47, was reduced significantly compared with that of unmethylated nick DNA (Fig. 6F and G). This result indicates that methylation near the nick site inhibits the kink formation and thus increases the stiffness of nick DNA. Methylation seems to increase the stability of base pairing and thus the melting temperature is increased locally near the methylation site. When the methylation site was 5 bp or 10 bp from the nick, it did not inhibit the kink formation of the nick region. Therefore, these results indicate that methylation in the kink region increases the local melting temperature of dsDNA in Fig. 4, which suppresses the formation of a kink and makes dsDNA stiffer.

## Conclusions

**Fig. 7:**
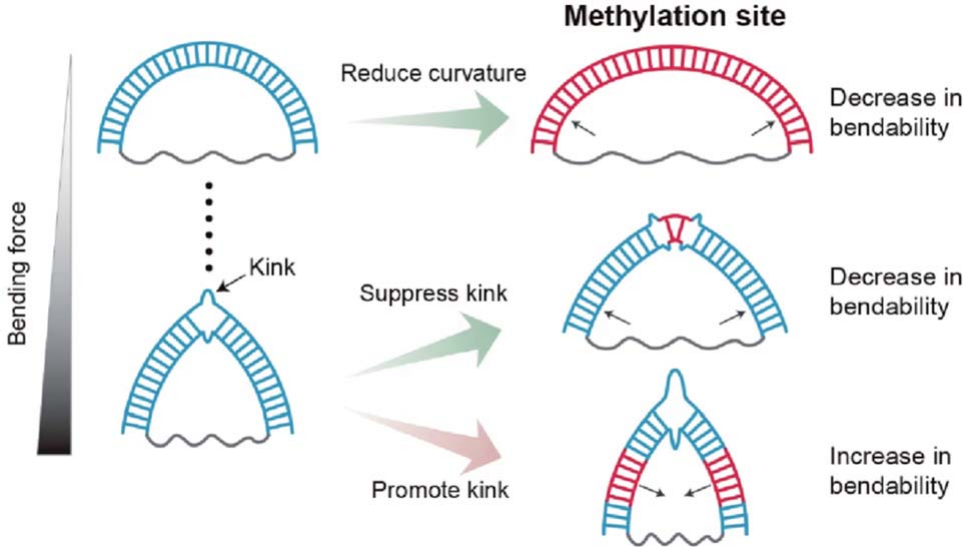
Schematic Illustrations of the regulatory effects of methylation on dsDNA bendability. Methylation sites and the kink of dsDNA are shown as the blue lines and a broken double-stranded structure, respectively. Methylation increases the stiffness of the DNA duplex under a weak bending force. Under a strong bending force, methylation at the non-kink region promotes kink formation, *i.e.*, dsDNA bendability increases. In contrast, methylation at the kink region suppresses kink formation, *i.e.*, dsDNA bendability decreases.

In this work, we observed that methylation has contrasting effects on the stiffness of dsDNA depending on DNA curvature and methylation location (Fig. 7). When dsDNA is under a weak bending force, methylation increases the stiffness of dsDNA, *i.e.*, the flexibility of dsDNA decreases. However, when dsDNA is bent by a strong force, dsDNA has a deformed kink structure.

In a kink state, methylation makes a kink form more favorable compared with a non-kink form, and thus, dsDNA become apparently more flexible. In contrast, when the methylation site is located in the kink region, the kink form is destabilized, and the bendability of dsDNA decreases.

Using short dsDNA, we showed that methylation increases the intrinsic stiffness of weakly bent dsDNA (Fig. 2). This result is in good agreement with the DNA looping time analysis, which showed that the looping time of 90 bp dsDNA increases as a function of the number of methylated sites.^19^ In contrast, measurements using optical and magnetic tweezers showed that methylation reduces the end-to-end distance of dsDNA under weakly stretching conditions.^22,23,59^ The differences in the dsDNA length and DNA sequences can contribute to the different bendabilities of dsDNA depending on the method.

Previous single-molecule FRET measurements showed that methylation decreases the stability of nucleosomes.^19^ In line with this study, it has been reported that methylation increases the structural energy of nucleosomes, especially when they are placed on minor grooves facing the histone core.^60^ However, other studies reported that the methylated sequence wraps around the histone core more tightly^36,37^ and that the increase in the density of methylated CpG islands is positively correlated with nucleosome occupancy.^16^ Thus, it is difficult to explain these seemingly contradictory results of methylation in the context of nucleosome stability.^61^ Although various factors may contribute to the stability of nucleosomes, our work shows that the variation in DNA mechanics with methylation can be one of the factors that regulates nucleosome stability (Fig. S9†). In the nucleosome, kinks, which include the breaking of the base-stacking interaction, are observed in the minor grooves facing the histone core.^62,63^ The kink in a D-shaped DNA is formed by base-pair melting and breaking base-stacking interaction.^31^ Thus, the kink in D-shaped DNA is not same as that in nucleosome. However, we showed that methylation at the kink site inhibits kink formation by stabilizing the local base-pair at the methylation site (Fig. 4–6). Thus, it is possible that the methylation at the kink region in the nucleosome also inhibits kink formation by stabilizing the local base-pair at the methylation site and thus destabilizes the nucleosome. The methylation site at the kink region may be one of the sources for nucleosome destabilization (Fig. S9B†). On the other hand, if the methylation site does not overlap with the kink region, kink formation may be favorable for stabilizing the bent dsDNA form, and thus nucleosomes may become more stable (Fig. S9C†).^11,54^ As a consequence, our result showing that methylation has different effects on DNA mechanics depending on its curvature and methylation location provides a clue to reconcile the contradictory observations that methylation increases or decreases the stability of nucleosome structure.

## Experimental section

### DNA sample preparation

We prepared ssDNA rings (methylated: Ring 38-M15, Ring 38-M22 and Ring 38-M15&17, unmethylated: Ring 34, Ring 38, Ring 40, Ring 52, Ring 52/CG 57%, Ring 52/CG67%, Ring 56, Ring 64, and Ring 100), a 48 nt linear ssDNA (Linear 48) and the complementary linear ssDNAs (Comp 30, Comp 30/CG 57%, Comp 30/CG 67%, Comp 30-M7, Comp 30-M16, Comp 30/CG 57%, Comp 30/CG 67%, Comp 30-M20, Comp 30-M25, Comp 30-M7&25, Comp 30-M16&20, Comp 30/CG 57% and Comp 30/CG 67%) for single-molecule FRET measurements and melting temperature measurements (Table S1†). We used the sequences of dsDNA portion, which were randomly designed before.^31^ To reduce the effects of the secondary structures of ssDNA strings, the ssDNA ring samples (Ring 52, Ring 56, Ring 64, and Ring 100) were designed to have a poly-dT sequences at the ssDNA string portions. The phosphorylation (/5Phos/) was added to the 5′ end of the ring ssDNA for cyclization and ligation. An amino modifier C6 dT (/iAmMC6T/) was added to the ring-ssDNA, linear-ssDNA, and complementary linear-ssDNA, for dye labeling. All oligomers were synthesized from Integrated DNA Technologies. We used ATTO 550 NHS-ester (AD550-31, ATTO-TEC) to label ring ssDNAs, and ATTO 647N NHS-ester (AD647N-31, ATTO-TEC) to label complimentary linear ssDNAs. The denaturing PAGE gel was used to purify the labeled ssDNAs. To form ssDNA ring, we annealed the ring ssDNA and partially complementary linear ssDNA (16 nt) which consists of each 8 nt complementary sequences for both ends of the ring ssDNA. Next, we used T4 ligase (2011A, TAKARA) to ligate both ends of the ring ssDNA. Using denaturing PAGE gels, ring forms of ssDNA were purified from un-ligased ssDNA and dimerized or tetramerized ring ssDNA, *etc.* We annealed the ssDNA ring and its complimentary linear ssDNA to form D-shaped DNA (Fig. 1A). The linear ssDNA and its complimentary linear ssDNA were annealed to form the linear dsDNA as a control. The nicked D-shaped DNA was prepared by annealing un-ligased ring ssDNA (Ring 52) and its complimentary linear ssDNA. All D-shaped DNA samples were stored in a buffer of 20 mM Tris–HCl (pH 8.0) and 400 mM NaCl.

### Calculation of the FRET efficiency and stoichiometry from smFRET measurements

We used the alternating laser excitation (ALEX) method to measure single-molecule FRET, which has been well described in previous work (Fig. S1†).^31^ Two alternating lasers for the donor excitation (532 nm laser; Samba-100, Cobolt) and the acceptor excitation (633 nm laser; 25-LHP-925, Melles-Griot) were used for ALEX. The alternations of the lasers were generated by the acousto-optic modulators (23080-1, Gooch & Housego) at a 100 μs period. A dichroic mirror (z532bcm, Chroma) was used to couple two laser lights. Before entering the microscope, the intensities of the donor and acceptor excitation lasers were 80 μW and 25 μW, respectively. The coupled laser beams were reflected from a dichroic mirror (Z532/633RPC, Chroma) inside an inverted microscope (IX 51, Olympus) and then focused on the 30 μm above the coverslip surface with an objective lens (UPLAPO 60X, Olympus). When a freely-diffusing DNA went through the focal volume, it was excited by the two lasers. The fluorescence was collected through the objective lens and passed through a 100 μm diameter pinhole. The beam splitter (625DCLP, Chroma) separated the fluorescence signals into two pathways, and these two fluorescence signals were refocused onto each avalanche photodiode (SPCM-AQRH-14, Excelitas technologies). The emissions of ATTO 550 and ATTO 647N were detected by two avalanche photodiode, respectively. Two filters (HQ580/60m and HQ660LP, Chroma) were used to reduce background noise of the donor emission and the acceptor emission, respectively.

The imaging buffer (20 mM Tris–HCl pH 8.0, 100 mM NaCl, 5% glycerol (v/v), 1 mM MEA, 0.01% BSA, and 2 mM MgCl_2_) was used to dilute samples to 50 pM. A burst of fluorescent signal in the time trace was produced by the in-and-out event of a molecule through the confocal volume. We selected the fluorescent bursts when the total photon-count number was higher than 30 per millisecond. In the ALEX method, a single burst includes three types of fluorescent intensities (*I*^A^_D_: the intensity of acceptor fluorescence by donor excitation laser, *I*^D^_D_: the intensity of donor fluorescence by donor excitation laser and *I*^A^_A_: the intensity of acceptor fluorescence by acceptor excitation laser). We corrected these fluorescent intensities using ratio of the detection channel efficiency (gamma factor), the donor emission detected by acceptor detection channel (leakage), and the emission of an acceptor excited directly by donor excitation laser (direct excitation), as previously described.^39^ The FRET efficiency, reporting distance between donor and acceptor dye, and stoichiometry, reporting the labeling status, were calculated from the corrected fluorescent intensities as follows,^38^
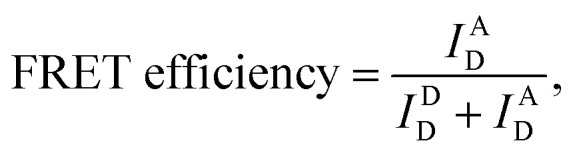

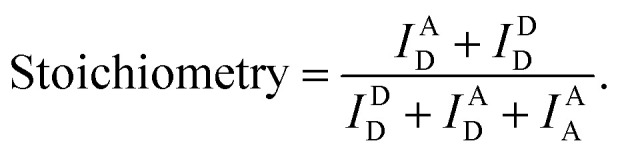


We collected the bursts and obtained the two-dimensional FERT efficiency-stoichiometry histogram. The burst cluster at the stoichiometry value close to 1 indicates donor-only species (Fig. S1C, the green box†), while the burst cluster at the stoichiometry value close to 0 indicates acceptor-only species (Fig. S1C, the red box†). We selected the bursts that were within the range of 0.2 < stoichiometry < 0.8 (Fig. S1C, the orange box†), which were used to obtain one-dimensional FRET efficiency histogram. The FRET measurements were performed at room temperature. The data acquisition and data analysis were performed using a home-built program (LABVIEW 8.6, National Instrument).

### High resolution melting curve analysis

We prepared linear dsDNAs by annealing un-ligased ring ssDNA and its complimentary linear ssDNA: unmethylated dsDNA (Ring 38 + Comp 30), dsDNA with a methylation site (Ring 38-M15 + Comp 30), dsDNA with two methylation sites (Ring 38-M15 + Comp 30-M16), and dsDNA with three methylation sites (Ring 38-M15&17 + Comp 30-M16). The high resolution melting curve was obtained from a 20 μL DNA sample solution (20 mM Tris–HCl pH 8.0, 100 mM NaCl, 2 mM MgCl_2_, 1x EvaGreen (31000-B500, Biofact), and 1 μM DNA sample) using real time PCR System (4376357, StepOne Real-Time PCR System, Applied Biosystems).^64^ The fluorescence data were collected between 60 °C and 95 °C in 0.1 °C increment with 15 s holding time. The melting temperature was obtained from the maximum value of the differential normalized fluorescence intensity curve (StepOne software ver. 2.1).

### The bending energy calculation from the simulation

The Monte Carlo simulation was used to obtain the equilibrium state of the semiflexible loop as described in previous work.^51^ The loop consisting of 38 connected nodes was used to create a coarse-grained model that represents the D-shaped DNA with 8 nt ssDNA string (D30-S8). Two types of nodes were used; 30 rigid nodes (1 ≤ *i* ≤ 30) representing the base pairs of dsDNA portion and 8 flexible nodes (31 ≤ *i* ≤ 38) representing the nucleotides of ssDNA string. The interval between the rigid nodes and the flexible nodes were set to be *a*_ds_ = 0.34 nm and *a*_ss_ = 0.7 nm, respectively (Fig. S6 and S7†). The bending energy of the *i*^th^ node (*E*_*i*_) was described by discrete worm-like chain model as follows,
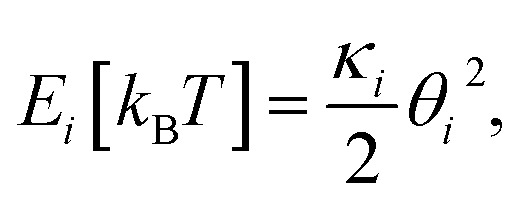
where *κ*_*i*_ is the bending modulus of the *i*^th^ node and *θ*_*i*_ is the angle between two adjacent tangential vectors of the *i*^th^ node. We set the bending modulus of rigid nodes as *L*_Pds_/*a*_ds_, where *L*_Pds_ is the persistence length of dsDNA, 50 nm, and the bending modulus of flexible nodes as *L*_Pss_/*a*_ss_, where *L*_Pss_, 3 nm, is the persistence length of ssDNA.^47^ Each step of simulation was performed using the pivotal moving of a randomly selected node. According to the rules of the Metropolis procedure,^49^ the trial structure after the pivotal moving was accepted or denied. The simulation of all nodes distributed in a circular form was equilibrated by 2 × 10^7^ MC steps. After achieving equilibration, the simulation was performed for 2 × 10^7^ MC steps and the bending energy of the rigid portion (
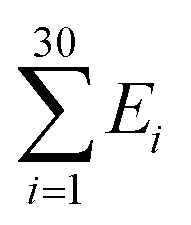
), *i.e.*, dsDNA portion, and the distance between 4^th^ and 27^th^ rigid nodes representing the dye-to-dye distance were recorded. All Monte Carlo simulations were performed using a handmade code (MATLAB 2019b, MathWorks).

## Data availability

The data found in this study are available within the main text and in the ESI.†

## Author contributions

S. Y., J. H., S. K. K., and N. K. L. designed the experiments. S. Y. and J. Y. prepared the DNA samples. S. Y., J. H., and J. Y. performed experiments. C. K. helped analysis. S. Y. performed the simulation and analytical calculation. S. Y. and N. K. L. wrote the manuscript. S. K. K. and N. K. L. supervised the project.

## Conflicts of interest

There are no conflicts of interest to declare.

## Supplementary Material

SC-013-D1SC07115G-s001

## References

[cit1] Doerfler W. (1983). Annu. Rev. Biochem..

[cit2] Law J. A., Jacobsen S. E. (2010). Nat. Rev. Genet..

[cit3] Riggs A. D. (1975). Cytogenet. Genome Res..

[cit4] Smith Z. D., Meissner A. (2013). Nat. Rev. Genet..

[cit5] Maunakea A. K., Nagarajan R. P., Bilenky M., Ballinger T. J., D'Souza C., Fouse S. D., Johnson B. E., Hong C., Nielsen C., Zhao Y., Turecki G., Delaney A., Varhol R., Thiessen N., Shchors K., Heine V. M., Rowitch D. H., Xing X., Fiore C., Schillebeeckx M., Jones S. J. M., Haussler D., Marra M. A., Hirst M., Wang T., Costello J. F. (2010). Nature.

[cit6] Bird A. P. (1986). Nature.

[cit7] Elango N., Yi S. V. (2011). Genetics.

[cit8] Patil V., Ward R. L., Hesson L. B. (2014). Epigenetics.

[cit9] Chen L., Chen K., Lavery L. A., Baker S. A., Shaw C. A., Li W., Zoghbi H. Y. (2015). Proc. Natl. Acad. Sci..

[cit10] Guo J. U., Su Y., Shin J. H., Shin J., Li H., Xie B., Zhong C., Hu S., Le T., Fan G., Zhu H., Chang Q., Gao Y., Ming G.-l., Song H. (2014). Nat. Neurosci..

[cit11] Pérez A., Castellazzi C. L., Battistini F., Collinet K., Flores O., Deniz O., Ruiz M. L., Torrents D., Eritja R., Soler-López M., Orozco M. (2012). Biophys. J..

[cit12] Severin P. M. D., Zou X., Gaub H. E., Schulten K. (2011). Nucleic Acids Res..

[cit13] Basu A., Bobrovnikov D. G., Qureshi Z., Kayikcioglu T., Ngo T. T. M., Ranjan A., Eustermann S., Cieza B., Morgan M. T., Hejna M., Rube H. T., Hopfner K.-P., Wolberger C., Song J. S., Ha T. (2021). Nature.

[cit14] Luger K., Mäder A. W., Richmond R. K., Sargent D. F., Richmond T. J. (1997). Nature.

[cit15] Lee J. Y., Lee J., Yue H., Lee T.-H. (2015). J. Biol. Chem..

[cit16] Collings C. K., Anderson J. N. (2017). Epigenet. Chromatin.

[cit17] Basu A., Bobrovnikov D. G., Ha T. (2021). J. Mol. Biol..

[cit18] Nathan D., Crothers D. M. (2002). J. Mol. Biol..

[cit19] Ngo T. T. M., Yoo J., Dai Q., Zhang Q., He C., Aksimentiev A., Ha T. (2016). Nat. Commun..

[cit20] Cassina V., Manghi M., Salerno D., Tempestini A., Iadarola V., Nardo L., Brioschi S., Mantegazza F. (2016). Biochim. Biophys. Acta, Gen. Subj..

[cit21] Wanunu M., Cohen-Karni D., Johnson R. R., Fields L., Benner J., Peterman N., Zheng Y., Klein M. L., Drndic M. (2011). J. Am. Chem. Soc..

[cit22] Pongor C. I., Bianco P., Ferenczy G., Kellermayer R., Kellermayer M. (2017). Biophys. J..

[cit23] Shon M. J., Rah S.-H., Yoon T.-Y. (2019). Sci. Adv..

[cit24] Yang Y.-J., Dong H.-L., Qiang X.-W., Fu H., Zhou E.-C., Zhang C., Yin L., Chen X.-F., Jia F.-C., Dai L., Tan Z.-J., Zhang X.-H. (2020). J. Am. Chem. Soc..

[cit25] Zaichuk T., Marko J. F. (2021). Biophys. J..

[cit26] Tanaka H., Sato S., Koyama M., Kujirai T., Kurumizaka H. (2020). J. Biochem..

[cit27] Shroff H., Reinhard B. M., Siu M., Agarwal H., Spakowitz A., Liphardt J. (2005). Nano Lett..

[cit28] Shroff H., Sivak D., Siegel J. J., McEvoy A. L., Siu M., Spakowitz A., Geissler P. L., Liphardt J. (2008). Biophys. J..

[cit29] Qu H., Wang Y., Tseng C.-Y., Zocchi G. (2011). Phys. Rev. X.

[cit30] Lee O. C., Kim C., Kim J. Y., Lee N. K., Sung W. (2016). Sci. Rep..

[cit31] Kim C., Lee O. C., Kim J. Y., Sung W., Lee N. K. (2015). Angew. Chem., Int. Ed..

[cit32] Yeou S., Lee N. K. (2021). Bull. Korean Chem. Soc..

[cit33] Ha T., Enderle T., Ogletree D. F., Chemla D. S., Selvin P. R., Weiss S. (1996). Proc. Natl. Acad. Sci..

[cit34] Fields A. P., Meyer E. A., Cohen A. E. (2013). Nucleic Acids Res..

[cit35] Park G., Cho M. K., Jung Y. (2021). J. Chem. Theory Comput..

[cit36] Lee J. Y., Lee T.-H. (2012). J. Am. Chem. Soc..

[cit37] Choy J. S., Wei S., Lee J. Y., Tan S., Chu S., Lee T.-H. (2010). J. Am. Chem. Soc..

[cit38] Kapanidis A. N., Lee N. K., Laurence T. A., Doose S., Margeat E., Weiss S. (2004). Proc. Natl. Acad. Sci..

[cit39] Lee N. K., Kapanidis A. N., Wang Y., Michalet X., Mukhopadhyay J., Ebright R. H., Weiss S. (2005). Biophys. J..

[cit40] Hellenkamp B., Schmid S., Doroshenko O., Opanasyuk O., Kühnemuth R., Rezaei Adariani S., Ambrose B., Aznauryan M., Barth A., Birkedal V., Bowen M. E., Chen H., Cordes T., Eilert T., Fijen C., Gebhardt C., Götz M., Gouridis G., Gratton E., Ha T., Hao P., Hanke C. A., Hartmann A., Hendrix J., Hildebrandt L. L., Hirschfeld V., Hohlbein J., Hua B., Hübner C. G., Kallis E., Kapanidis A. N., Kim J.-Y., Krainer G., Lamb D. C., Lee N. K., Lemke E. A., Levesque B., Levitus M., McCann J. J., Naredi-Rainer N., Nettels D., Ngo T., Qiu R., Robb N. C., Röcker C., Sanabria H., Schlierf M., Schröder T., Schuler B., Seidel H., Streit L., Thurn J., Tinnefeld P., Tyagi S., Vandenberk N., Vera A. M., Weninger K. R., Wünsch B., Yanez-Orozco I. S., Michaelis J., Seidel C. A. M., Craggs T. D., Hugel T. (2018). Nat. Methods.

[cit41] Hwang J., Kim J.-Y., Kim C., Park S., Joo S., Kim S. K., Lee N. K. (2020). eLife.

[cit42] Evans G. W., Hohlbein J., Craggs T., Aigrain L., Kapanidis A. N. (2015). Nucleic Acids Res..

[cit43] Fijen C., Mahmoud M. M., Kronenberg M., Kaup R., Fontana M., Towle-Weicksel J. B., Sweasy J. B., Hohlbein J. (2020). J. Biol. Chem..

[cit44] Thompson R. E., Larson D. R., Webb W. W. (2002). Biophys. J..

[cit45] Chuang H.-M., Reifenberger J. G., Cao H., Dorfman K. D. (2017). Phys. Rev. Lett..

[cit46] Baczynski K., Lipowsky R., Kierfeld J. (2007). Phys. Rev. E: Stat., Nonlinear, Soft Matter Phys..

[cit47] Murphy M. C., Rasnik I., Cheng W., Lohman T. M., Ha T. (2004). Biophys. J..

[cit48] Smith S. B., Cui Y., Bustamante C. (1996). Science.

[cit49] Metropolis N., Rosenbluth A. W., Rosenbluth M. N., Teller A. H., Teller E. (1953). J. Chem. Phys..

[cit50] Le T. T., Kim H. D. (2014). Nucleic Acids Res..

[cit51] Jeong J., Kim H. D. (2019). Phys. Rev. Lett..

[cit52] Zheng X., Vologodskii A. (2009). Biophys. J..

[cit53] Nardo L., Lamperti M., Salerno D., Cassina V., Missana N., Bondani M., Tempestini A., Mantegazza F. (2015). Nucleic Acids Res..

[cit54] Song Q., Qiu Z., Wang H., Xia Y., Shen J., Zhang Y. (2013). Struct. Chem..

[cit55] Wang J., Pan X., Liang X. (2016). J. Anal. Methods Chem..

[cit56] Protozanova E., Yakovchuk P., Frank-Kamenetskii M. D. (2004). J. Mol. Biol..

[cit57] Yakovchuk P., Protozanova E., Frank-Kamenetskii M. D. (2006). Nucleic Acids Res..

[cit58] Cong P., Dai L., Chen H., van der Maarel Johan R. C., Doyle P. S., Yan J. (2015). Biophys. J..

[cit59] Zaichuk T., Marko J. F. (2021). Biophys. J..

[cit60] Portella G., Battistini F., Orozco M. (2013). PLoS Comput. Biol..

[cit61] Jimenez-Useche I., Ke J., Tian Y., Shim D., Howell S. C., Qiu X., Yuan C. (2013). Sci. Rep..

[cit62] Richmond T. J., Davey C. A. (2003). Nature.

[cit63] Ong M. S., Richmond T. J., Davey C. A. (2007). J. Mol. Biol..

[cit64] Wojdacz T. K., Dobrovic A., Hansen L. L. (2008). Nat. Protoc..

