# Supplementary material for: Cytosine methylation regulates DNA bendability depending on the curvature
Source: Chem Sci. 2022 Jun 2;13(25):7516–25. doi: 10.1039/d1sc07115g (PMC9242020; doi:10.1039/d1sc07115g)
Supplement: SC-013-D1SC07115G-s001 [file SC-013-D1SC07115G-s001.pdf]

Electronic Supplementary Material (ESI) for Chemical Science.
This journal is © The Royal Society of Chemistry 2022

Supplementary Information for

"Cytosine methylation regulates DNA bendability depending on the curvature"

Sanghun Yeou[‡,a], Jihee Hwang[‡,a], Jaehun Yi[a], Cheolhee Kim[b], Seong Keun Kim[a], and Nam Ki Lee[*,a]

[a]Department of Chemistry, Seoul National University, 08832, Seoul, Republic of Korea

[b]National Science Museum, Daejeon, 34143, Republic of Korea

[‡] These authors contributed equally to this work

* Email: namkilee@snu.ac.kr

Calculation of the relative stiffness of methylated dsDNA

We obtained the average dye-to-dye distance [$R_{dye} = R_0(1/E - 1)^{1/6}$] of DNA samples using the average FRET efficiency (E) and the Förster radius of the ATTO 550-ATTO 647N pair (R_0 = 6.5 nm). Because the bending angle of the dsDNA sample is small in a weakly bent form, we assumed the end-to-end distance of dsDNA portion as $R = 5R_{dye}/4$ in order to simplify the calculation. The elastic rod, such as a short dsDNA, resists to a compression and remains a straight form until the bending force (F_{bend}) is larger than the critical force (F_C). The critical force is proportional to the stiffness (κ). When the compressive force is slightly larger than the critical force, the linear approximation of the relative force (F_{bend} /F_C) and the relative end-to-end distance (R/L_{ds}), where L_{ds} is a contour length of dsDNA, are given by,[1]

$$1 - \frac{R}{L_{ds}} \approx 2\left(\frac{F_{bend}}{F_C} - 1\right).$$

In a D-shaped DNA nanostructure, dsDNA portion is compressed by the stretching force of a ssDNA string (F_{st}). The stretching force is modeled by worm-like chain[2] as follows,

$$F_{st} = \frac{k_B T}{L_{Pss}}\left(\frac{1}{4\left(1 - R/L_{ss}\right)^2} - \frac{1}{4} + \frac{R}{L_{ss}}\right),$$

where L_{ss} denotes the contour length of ssDNA string and L_{Pss} (= 3 nm) is the persistence length of ssDNA.[3] At the equilibrium state, the bending force is obtained from the relation, $F_{st} = F_{bend}$.

The FRET efficiency of the D-shaped DNA with 70 nt ssDNA string (D30-S70) was slightly higher than the FRET efficiency of the linear dsDNA. This result suggests that the compressive force for the dsDNA portion of D30-S70 is slightly higher than the critical force. Thus, we obtained the reduced end-to-end distance $(\Delta R = |R - R_{D30-S70}|)$ and the force increment $(\Delta F_{bend} = |F_{bend} - F_{D30-S70}|)$ of D30-S34, D30-S26, and D30-S22, as described in fig. 2D. We can obtain equation as follows,

$$\Delta R = \frac{2L_{ds}}{F_C}\Delta F_{bend} \propto \left(\frac{1}{\kappa}\right)\Delta F_{bend}.$$

Using this equation, we obtained the relative stiffness of dsDNA by comparing the slope of linear approximation using Origin 8.5.

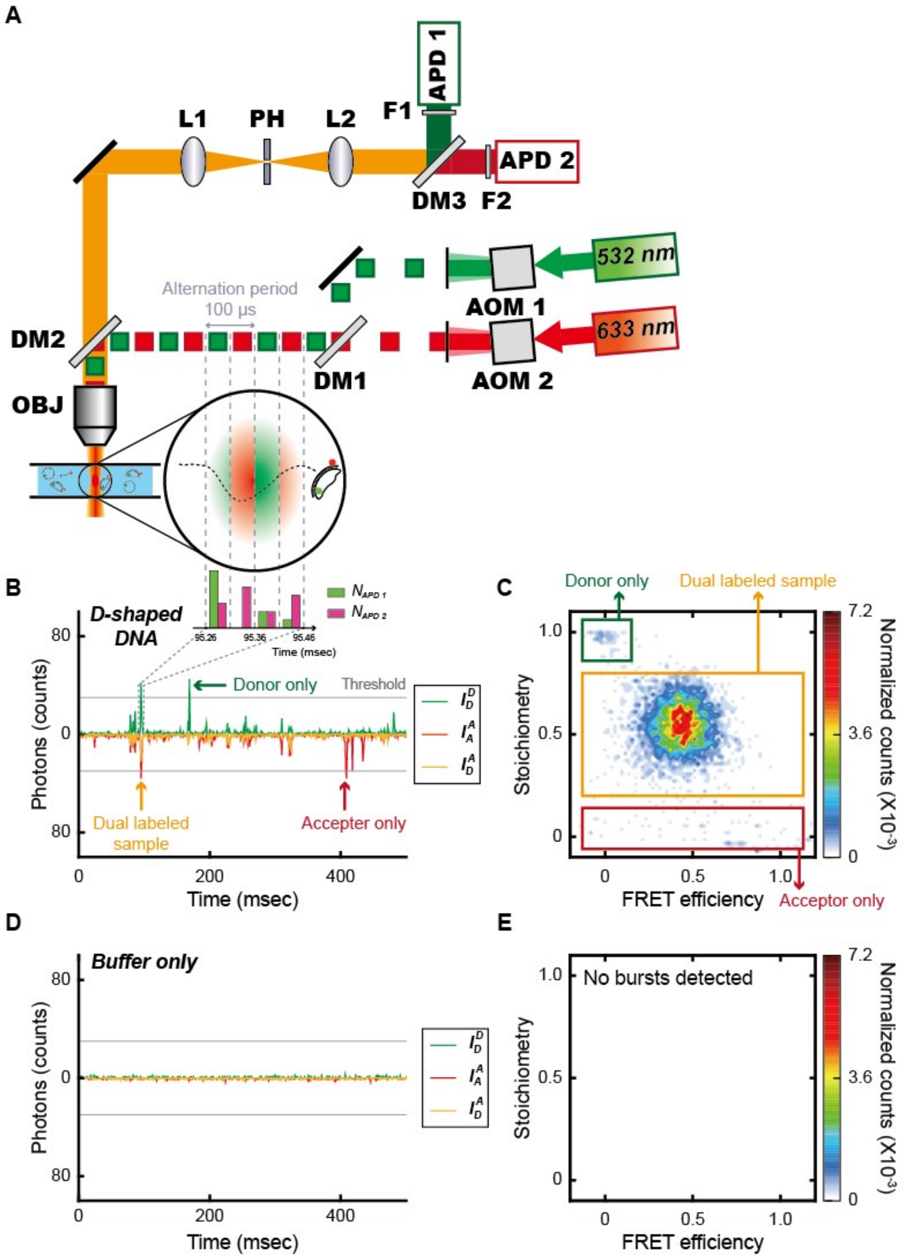

Fig. S1 Brief description of single-molecule alternating laser excitation setup. (A) Schematic illustration of single-molecule microscopy setup with alternating laser excitation. AOM: acoustic optical modulator, DM: dichroic mirror, OBJ: objective lens, L: lens, PH: pinhole, F: filter, and APD: avalanche photodiode (single photon counting module). The two lasers (532 nm and 633 nm) were used with an alternation period 100 µs. (B) Time traces of D-shaped DNA sample obtained from ALEX measurement. Photons were detected during a molecule passing through the confocal volume. $N_{APD\ 1}$ is the photon counting number of APD 1 and $N_{APD\ 2}$ is the photon counting number of APD 2. I_D^D is the fluorescent emission intensity of APD 1 excited by the 532 nm laser (donor

emission intensity), I_A^A the fluorescent emission intensity of APD 2 excited by the 633 nm laser (acceptor emission intensity), and I_D^A the fluorescent emission intensity of APD 2 excited by the 532 nm laser (FRET intensity). (C) A two-dimensional FRET efficiency-Stoichiometry histogram of D-shaped DNA sample was obtained from the fluorescence bursts in time traces. The green and red boxes denote donor-only and acceptor-only species, respectively. The orange box indicates the dual labeled species, i.e., FRET samples. (D) Time traces of buffer only sample obtained from ALEX measurement. (E) A two-dimensional FRET efficiency-Stoichiometry histogram of buffer only sample.

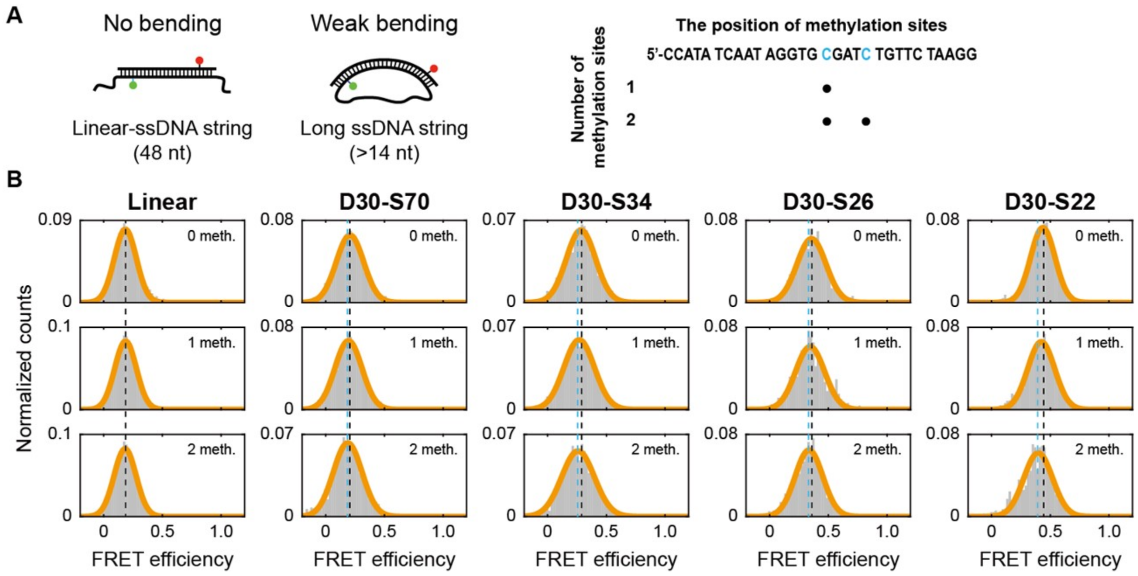

Fig. S2 1D FRET efficiency histogram for the linear and the D-shaped DNAs (D30-S70, D30-S34, D30-S26, and D30-S22) depending on the number of methylation sites. (A) Schematic representations of the linear DNA (no bending) and the D-shaped DNA with a low curvature (weak bending). D-shaped DNAs with ssDNA string lengths longer than 16 nt were used. The methylation sites on the dsDNA portion are shown in blue in the right panel. (B) 1D FRET histograms for linear DNA and the D-shaped DNA with a low curvature. The length of the ssDNA string was gradually reduced from 70 nt to 22 nt (D30-S70, D30-S34, D30-S26, and D30-S22). The histograms were fitted to a single Gaussian distribution. The average FRET efficiency of unmethylated sample and two methylated sample are denoted black and blue dot line, respectively.

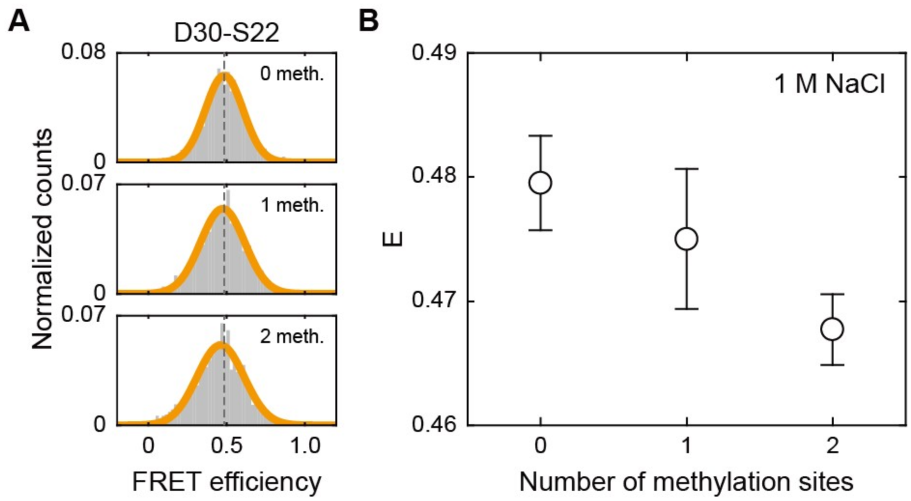

Fig. S3 The effect of methylation on dsDNA stiffness under a high salt condition (1 M NaCl). (A) 1D FRET histograms of D30-S22 depending on the number of methylation sites. Each histogram was fitted to a single Gaussian distribution. (B) The average FRET efficiencies (E) of D30-S22 depending on the number of methylation sites. The error bars were obtained from three independent measurements.

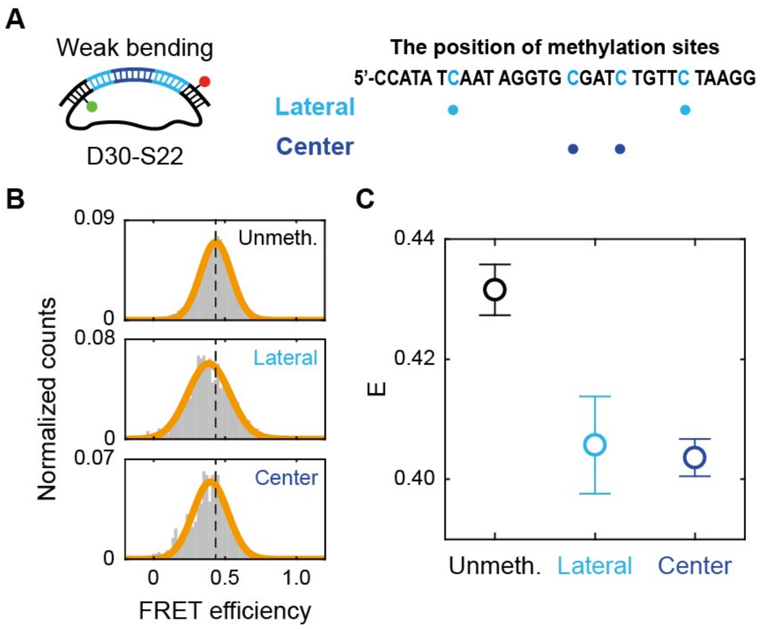

Fig. S4 The effect of methylation on dsDNA stiffness depending on the positions of methylation sites. (A) Schematic representation of D-shaped DNA (D30-S22). The methylation sites in the sequence are denoted by dots in the right panel. (B) 1D FRET histograms of D30-S22 with different methylation sites. Each histogram was fitted to a single Gaussian distribution. (C) The average FRET efficiencies (E) of the D30-S22 samples depending on the methylation sites. The error bars were obtained from three independent measurements.

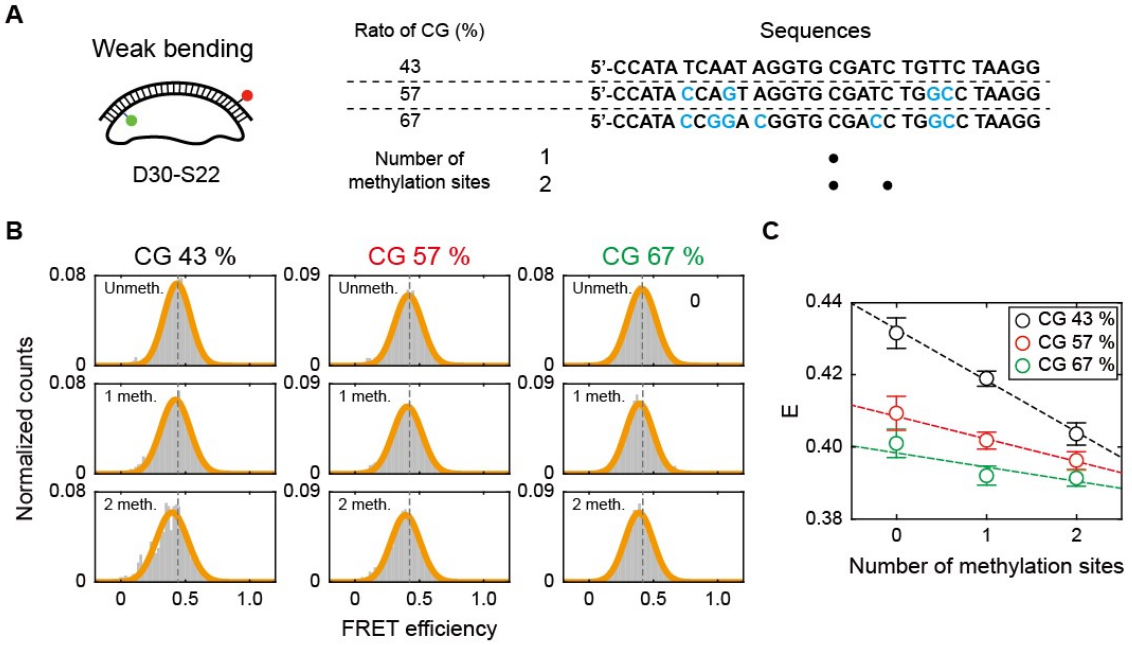

Fig. S5 The effect of methylation on dsDNA stiffness depending on the CG ratio. (A) Schematic representation of D-shaped DNA (D30-S22) with a low curvature (weak bending). The additional CG bases on the dsDNA portion are denoted by a blue color. (B) 1D FRET histograms of D30-S22 depending on the methylation number and the CG ratio. Each histogram was fitted to a single Gaussian distribution. (C) The FRET efficiencies (E) of D30-S22 depending on the number of methylation sites. The error bars were obtained from three independent measurements.

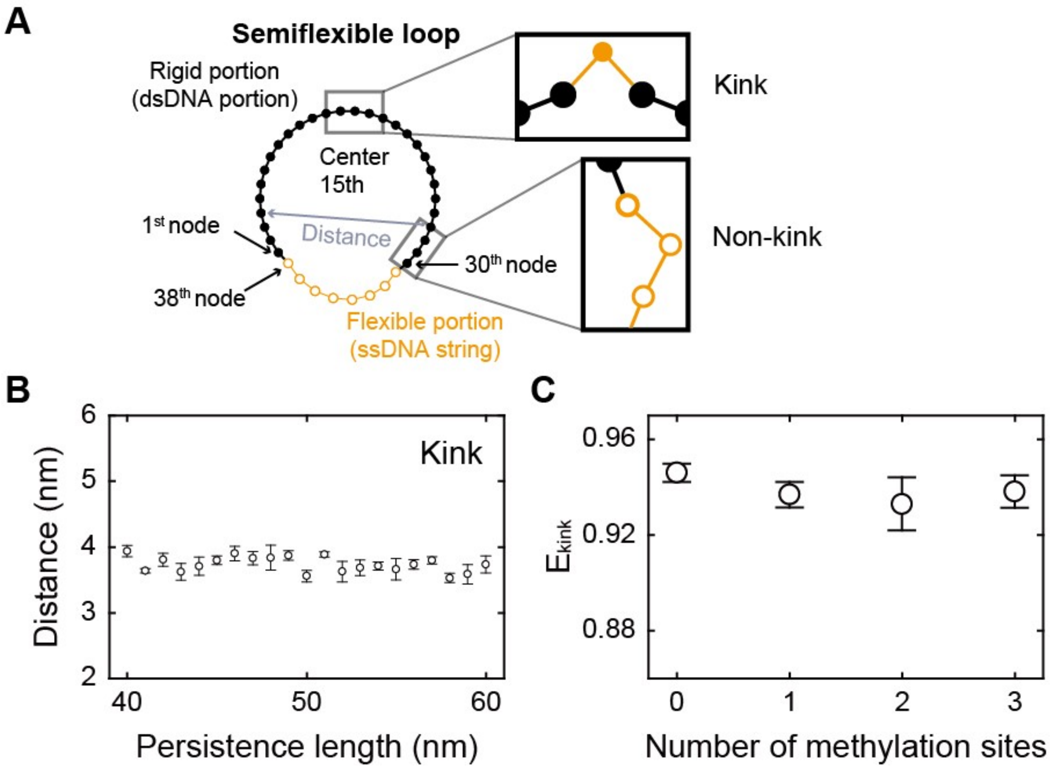

Fig. S6 Monte-Carlo simulation for the kink form: the dye-to-dye distance depending on the persistence length of dsDNA. (A) A brief illustration of a semiflexible loop used in the simulation to obtain the bending energy of dsDNA and the distance between the dye-labeled base pairs. The loop consists of two types of nodes: 30 rigid nodes (representing dsDNA base pairs) and 8 flexible nodes (representing ssDNA nucleotides). In the kink state, the node placed at the center of the rigid portion (15th node) becomes flexible. In the non-kink state, three nodes placed on both ends of the rigid portion become flexible. (B) The average distance between the 4th and the 27th nodes of the kink state as a function of the persistence length of rigid nodes, which was obtained from the MC simulation. The error bars were obtained from the five independent simulations. (C) The average FRET efficiencies of the kink state (E_{kink}) depending on the number of methylation sites, which were obtained from fig. 3B. The error bars were obtained from the three independent measurements.

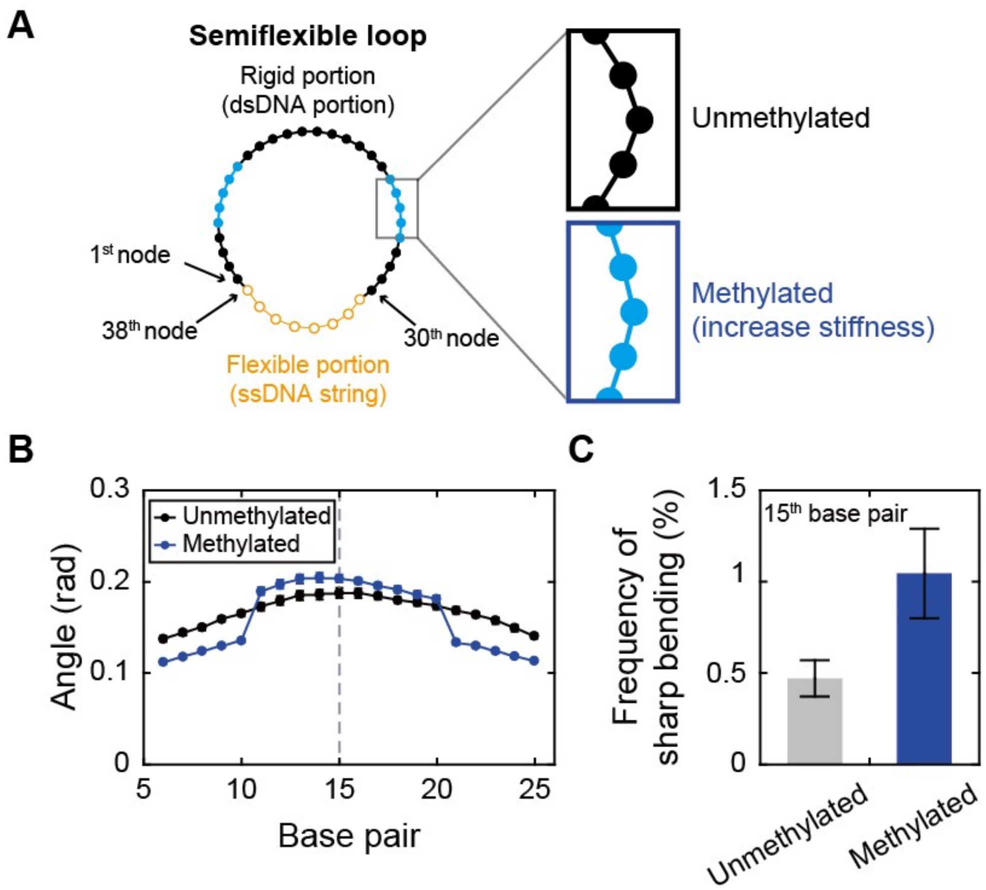

Fig. S7 Monte-Carlo simulation for the effect of the local stiffness on the flanking region of the dsDNA portion and the curvature at the central region of the dsDNA portion. (A) A brief illustration of a semiflexible loop used to obtain the bending angle of each node and the frequency of the sharp bending at the 15th node. The loop consists of two types of nodes: 30 rigid nodes (representing dsDNA base pairs) and 8 flexible nodes (representing ssDNA nucleotides). We set the methylated state to have stiffer nodes (the persistence length: 70 nm) placed on the flanking region of rigid portion (the 5th - 10th and 21th - 25th nodes). (B) The average angle of each node for the unmethylated and the methylated states was obtained from MC simulation. The error bars were obtained from the five independent simulations. (C) The frequency of the sharp bending (> 0.4 rad) of the node placed on the center (15th node). The error bars were obtained from the five independent simulations.

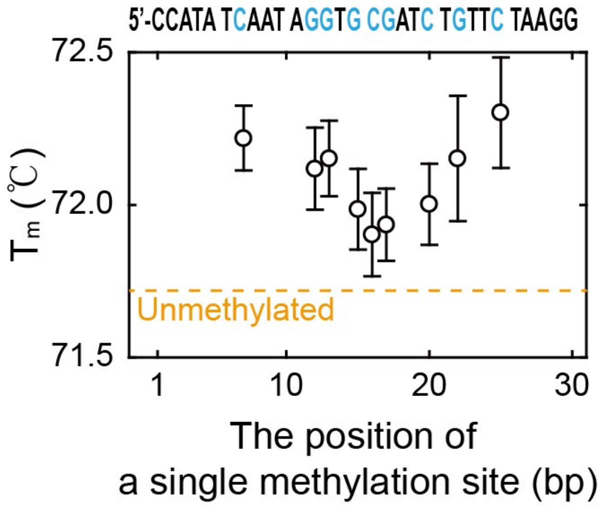

Fig. S8 Melting temperature of dsDNA depending on the position of a single methylation site. Methylation sites are denoted by the blue color in the sequence. Melting temperature of unmethylated dsDNA is shown as the yellow dotted line. The error bars were obtained from three independent measurements.

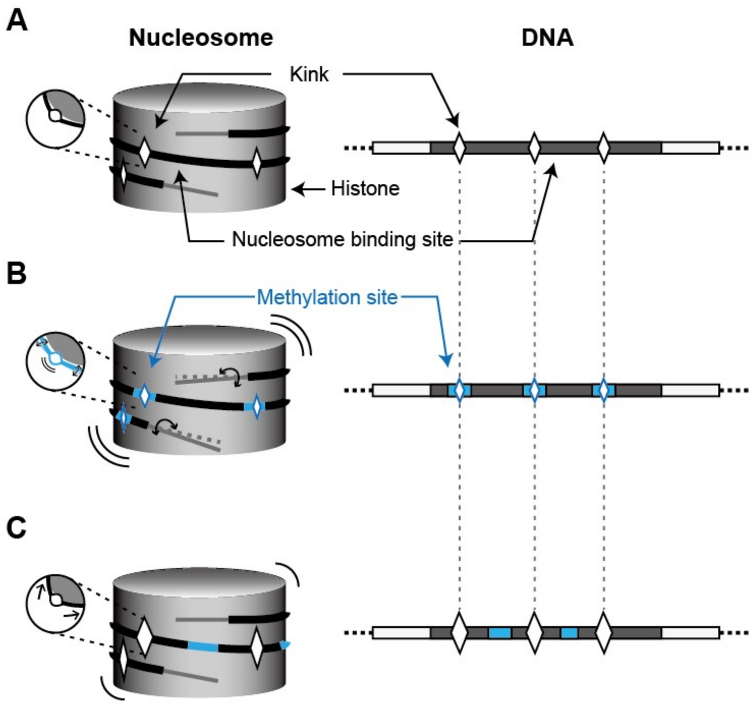

Fig. S9 (A) Brief illustration of the nucleosome. The kink regions are marked by open diamonds. (B) Methylation at kink sites suppresses kink formation, which will make nucleosomes unstable. (C) Methylation at the non-kink region promotes kink formation, which will make nucleosomes more stable.

Table S1 List of DNA sequences for smFRET measurements. ATTO 550 was labeled at /iAmMC6T/ of all sequences in ring ssDNA and linear ssDNA. ATTO 647N was labeled at /iAmMC6T/ of all sequences in complimentary linear ssDNA. The red colors in ring ssDNA indicate dsDNA portions of D-shaped DNAs.

**Ring ssDNA**	
Name	Sequences
Ring 34	/5Phos/TACCT/iAmMC6T/AGAACAGATCGCACCTATTGATATGGAT
Ring 38	/5Phos/TATACCT/iAmMC6T/AGAACAGATCGCACCTATTGATATGGATAT
Ring 38-M15	/5Phos/TATACCT/iAmMC6T/AGAACAGATCG/iMe-dC/ACCTATTGATATGGATAT
Ring 38-M22	/5Phos/TATACCT/iAmMC6T/AGAA/iMe-dC/AGATCGCACCTATTGATATGGATAT
Ring 38-M15&17	/5Phos/TATACCT/iAmMC6T/AGAACAGAT/iMe-dC/G/iMe-dC/ACCTATTGATATGGATAT
Ring 40	/5Phos/CTATACCT/iAmMC6T/AGAACAGATCGCACCTATTGATATGGATATT
Ring 52	/5Phos/CACCTATTGATATGGTTTTTTTTTTTTTTTTTTTTTCCT/iAmMC6T/AGAACAGATCG
Ring 52 / CG 57%	/5Phos/CACCTACTGGTATGGTTTTTTTTTTTTTTTTTTTTTCCT/iAmMC6T/AGGCCAGATCG
Ring 52 / CG 67%	/5Phos/CACCGTCCGGTATGGTTTTTTTTTTTTTTTTTTTTTCCT/iAmMC6T/AGGCCAGGTCG
Ring 56	/5Phos/CACCTATTGATATGGTTTTTTTTTTTTTTTTTTTTTTTTTCCT/iAmMC6T/AGAACAGATCG
Ring 64	/5Phos/CACCTATTGATATGGTTTTTTTTTTTTTTTTTTTTTTTTTTTTTTTTTCCT/iAmMC6T/AGAACAGATCG
Ring 100	/5Phos/CACCTATTGATATGGTTTTTTTTTTTTTTTTTTTTTTTTTTTTTTTTTTTTTTTTTTTTTTTTTTTTTTTTTTTTTTTTTTTTTTTTCCT/iAmMC6T/AGAACAGATCG
**Linear ssDNA**	
Name	Sequences
Linear 48	TTTTTTTTTCCT/iAmMC6T/AGAACAGATCGCACCTATTGATATGGTTTTTTTTT
**Complimentary linear ssDNA**	
Name	Sequences
Comp 30	CCA/iAmMC6T/ATCAATAGGTGCGATCTGTTCTAAGG
Comp 30 / CG 57%	CCA/iAmMC6T/ACCAGTAGGTGCGATCTGGCCTAAGG
Comp 30 / CG 67%	CCA/iAmMC6T/ACCGGACGGTGCGACCTGGCCTAAGG
Comp 30-M7	CCA/iAmMC6T/AT/iMe-dC/AATAGGTGCGATCTGTTCTAAGG
Comp 30-M16	CCA/iAmMC6T/ATCAATAGGTG/iMe-dC/GATCTGTTCTAAGG
Comp 30-M16 / CG 57%	CCA/iAmMC6T/ACCAGTAGGTG/iMe-dC/GATCTGGCCTAAGG
Comp 30-M16 / CG 67%	CCA/iAmMC6T/ACCGGACGGTG/iMe-dC/GACCTGGCCTAAGG

Comp 30-M20	CCA/iAmMC6T/ATCAATAGGTGCGAT/iMe-dC/TGTTCTAAGG
Comp 30-M25	CCA/iAmMC6T/ATAATAGGTGCGATCTGTT/iMe-dC/TAAGG
Comp 30-M7&25	CCA/iAmMC6T/AT/iMe-dC/AATAGGTGCGATCTGTT/iMe-dC/TAAGG
Comp 30-M16&20	CCA/iAmMC6T/ATCAATAGGTG/iMe-dC/GAT/iMe-dC/TGTTCTAAGG
Comp 30-M16 & M20 / CG 57%	CCA/iAmMC6T/ACCAGTAGGTG/iMe-dC/GAT/iMe-dC/TGGCCTAAGG
Comp 30-M16 & M20 / CG 67%	CCA/iAmMC6T/ACCGGACGGTG/iMe-dC/GAC/iMe-dC/TGGCCTAAGG

SI References

1. K. Baczynski, R. Lipowsky and J. Kierfeld, *Physical Review E*, 2007, **76**, 061914.
2. C. Kim, O. C. Lee, J. Y. Kim, W. Sung and N. K. Lee, *Angew Chem Int Ed Engl*, 2015, **54**, 8943-8947.
3. M. C. Murphy, I. Rasnik, W. Cheng, T. M. Lohman and T. Ha, *Biophysical Journal*, 2004, **86**, 2530-2537.
